# Preoptic and hypothalamic regulation of multi-tiered, chronologically arranged female rat sexual behavior

**DOI:** 10.1186/s12576-023-00890-4

**Published:** 2023-12-08

**Authors:** Yasuo Sakuma

**Affiliations:** Department of Anatomy and Neurobiology, Graduate School of Medical Sciences, Nippon Medical School, 25-16 Nezu 1 Chome, Tokyo, 113-8602 Japan

## Abstract

As in many mammalian behaviors, sexual behavior exhibits structure. Each modular components of the structure, that are linked together over time, occur in probabilistic manner. Endocrine milieu, in particular sex hormones, define the probability to synchronize the behavior with the production of gametes. Developmental experience and environmental cues affect the hormonal milieu of the brain. This is especially true in female mammals, in which ova mature with certain intervals along with ovarian secretion of sex hormones. Estrogens secreted by mature ovarian follicles support both affiliative and executive components of female sexual behavior. In the absence of the ovarian steroids, females avoid males when possible, or antagonize and reject males when put together. Female sexual behavior is intimately linked with the estrous cycle in many species such that females are only receptive for a brief period at the estrus stage surrounding ovulation. Thus, in the rat, females strongly influence the outcome of mating encounter with a male. Affiliative or solicitatory behavior shown by females in estrus leads to the female adapting the lordosis posture, which is characterized by hindleg postural rigidity and lordotic dorsiflexion of the spine, in response to touch-pressure somatosensory stimuli on the skin of the flanks, rump-tail base, perineum region given by male partner. The posture facilitates intromission and consequently fertilization. Although dependence on estrogens is the most important feature of female rat sexual behavior, cervical probing combined with palpation of the hindquarter skin acts as a supranormal stimulus to elicit lordosis. Thus, lordosis behavior is a hub of multi-tiered, chronologically arranged set of behaviors and estrogen appear to alter excitability of neural network for lordosis.

The lordosis reflex, dorsiflexion of the vertebral column (Fig. 1), is an essential element of female rat copulatory behavior. This stereotyped behavior depends strongly on estrogen and elicited by somatosensory stimuli on rump-tail base-perineal skin [1]. Odor cues influence female rats' mate choice of male partners [2, 3], but are not indispensable for the execution of the behavior [4]; the sighting of the particular male mating with another female do not necessarily alter females’ choice [5]. Ultrasound vocalization in the rat and mouse express their arousal and emotional states [6], however, is correlated with sexual motivation rather than the lordosis behavior [7].

**Fig. 1:**
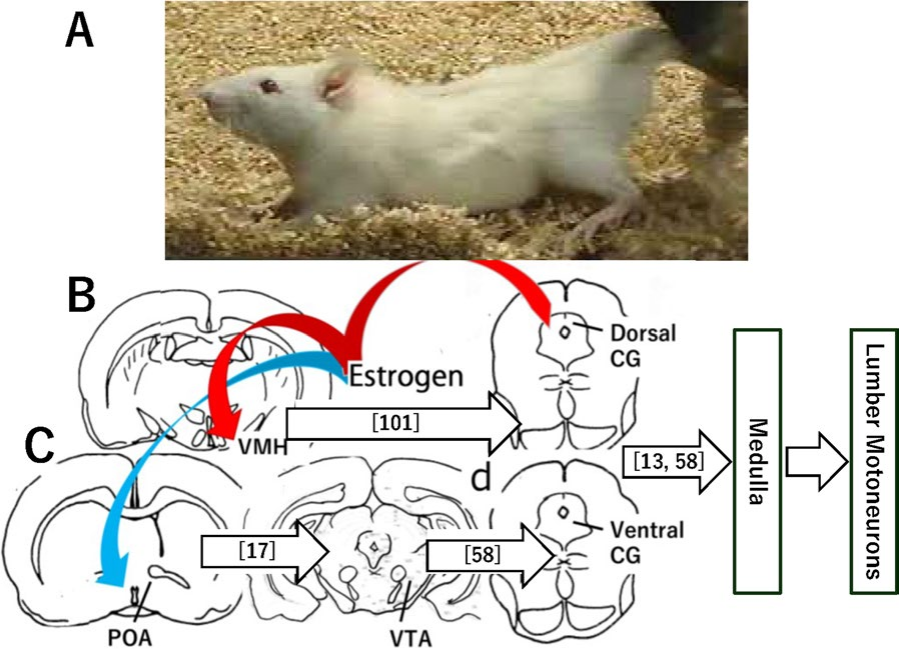
**A**. Lordosis posture in a female rat in estrus, immediately after the individual was dismounted; **B**. Projections from the ventromedial nucleus of the hypothalamus (VMH) or the medial preoptic area (POA). Arrows depict sites of estrogen induced excitation (**B**, in red) or inhibition (**C**, in blue). Other *CG* midbrain central gray, *VTA* ventral tegmental area. Reference number for each connection is in parentheses

## Neural axis for lordosis behavior

### Sites for estrogen action

An earlier study by Barfield and Chen [8] showed stereotaxic implants of crystalline estrogen (estradiol benzoate) were effective in both the preoptic area (POA) and the ventromedial hypothalamic nucleus (VMH), particularly, its ventrolateral quadrant (vlVMH), in terms of lordosis and preceding affiliative behaviors, such as ear wiggling and darting, with the most intense responses were induced by the implants in the VMH. Electrical stimulation study revealed crucial role of the VMH in supporting lordosis reflex in ovariectomized female rats treated with subthreshold dose of estrogen [9]. Progressive increase in lordosis performance followed electrical stimulation of the VMH with a delayed onset and the effect persisted after termination of current application.

In contrast to the tardive response to VMH stimulation, electrical stimulation of the midbrain central gray (CG) caused a prompt and transient facilitation of lordosis [10]. The CG is a projection target of the VMH [11, 12]. CG neurons send axons to the medullary core, a site of origin of medullospinal projection which implement lordosis by innervating spinal motoneurons. Antidromic action potentials were recorded from CG neurons in response to stimulation of the medulla.

Thus, neural axis for the estrogen-dependent behavioral activation of lordosis originates in the VMH and descends to the medulla via the CG. Stimulation of the VMH increased the frequency of successful antidromic propagation into the somatodendritic complex of certain CG cells showing excitatory nature of the VMH innervation of these CG cells [13]. Estrogen treatment of the ovariectomized rat had a similar effect on the antidromic propagation as the VMH stimulation. ERα-expressing neurons project to the CG [14]. Estrogen enhances depolarizing action of N-methyl-D-aspartate (NMDA) and other molecules on VMH neurons by inhibiting K^+^ currents [15].

### Inhibition of lordosis by POA projection to the midbrain

Along with the vlVMH, implants of crystalline estrogen in the medial POA also enhances lordosis and other affiliative behaviors albeit a larger dose of the hormone is needed [8]. The behavioral activation is, however, not a result of neural activation, but due to inhibition of POA efferents to the midbrain [16, 17]. Electrical stimulation of the POA reduced the probability of successful propagation into the somatodendritic complex in antidromic action potentials. Stimulation of the ventromedial nucleus of the hypothalamus (VMH) increased it. Conversely, electrolytic lesion of the POA facilitated, while VMH lesion reduced antidromic spike invasion.

### Excitatory VMH innervation of the CG

Electrical stimulation studies [9] suggest that estrogen in the vlVMH causes behavioral activation of lordosis while causes behavioral disinhibition in the POA. Partial subsistence of sexual behavior in female rats with vlVMH lesion [18] can be a result of estrogen action on the POA. Cholecystokinin A receptor-expressing cells in the vlVMH were the key controllers of female sexual behaviors [19]. Work in mice identified the vlVMH as the attack center. Ablation of ERα-positive vlVMH neurons in females greatly diminished sexual receptivity and in males reduced mating and aggression [20]. Yang et al*.* [21] showed that the activity of these vlVMH neurons in males represents aggression performed by self and others as mirror neurons. Thus, the vlVMH likely mediates multiple social behaviors and future studies will need to address how factors including social experience, hormonal state, and behavioral context influence which behaviors are generated.

In addition to classical ERα, the vlVMH contains neurons with a putative G-protein coupled membrane ER called GPR30 [22]. GPR30 binds 17β-estradiol with an affinity similar as ERα and activates both PKA and extracellular-regulated kinase signaling pathways. Administration of G-1, a selective agonist for GPR30 [23], in an estradiol-progesterone priming paradigm, increased lordosis behavior in female mice [24]. GPR30 activation phosphorylate the classical ERα, showing that crosstalk with ERα is important in the display of this and other behaviors, many of which are absent in ERα-null mice [25]. GPR30-mediated phosphorylation may be also involved in the estrogen-dependent masculinization of the POA during development [26].

### Progesterone and nonsteroidal molecules

Although the lordosis behavior can be elicited solely by estrogen in experimental settings, successive action of progesterone is needed to induce a full set of female rat sexual behavior. Estrogen induces progestin receptors or receptors for progesterone in the hypothalamus and preoptic area [27]. A series of pharmacological studies, initially conducted with substances such as GABA [28], gonadotropin releasing hormone (GnRH) [29, 30] and prostaglandin E2 [31] also modulated lordosis behavior in ovariectomized, estrogen-primed rats.

### Other neurotropic drugs

Neurotropic non-steroidal molecules, like reserpine, and other peptides, e.g., oxytocin, prolactin, opioids, substitutes progesterone, but not estrogen, to induce lordosis behavior. GABA may depolarize hypothalamic neurons due to high chloride content as we have shown in GnRH neurons [32]. The diversity of effective agents suggests that multiple signaling pathways may be involved in the regulation of female sexual behavior [33]. GABA action may be enhanced by a direct action of progesterone on GABA receptors [34].

The VMH is also important for the modulation of lordosis by molecules other than ovarian steroids, but, nevertheless, estrogen pretreatment is a prerequisite for these effects. Infusions of agonists or antagonists of acetylcholine [35], norepinephrine [36], and serotonin [37, 38] into the VMH have been examined with various results. Moreover, infusion of neuropeptides, such as GnRH [39], and oxytocin [40] into the VMH has been shown to alter lordosis. Oxytocin is one of non-steroidal molecules which has been shown in earlier studies to facilitate lordosis and affiliative behaviors in estrogen-primed ovariectomized rats [41, 42]. Affiliative responses in females constitute proceptive behavior toward male partners which in turn initiate copulatory interactions which include lordosis in females. Gentle tactile stimulation of rump, tail-base and perineum induces affiliative responses in female rats in estrus. Infusion of oxytocin antagonist into the medial POA increases rejection and disrupts receptivity in estrogen-primed ovariectomized rats [43]. Because transcription of both oxytocin ligand and its receptor depends on estrogen, observations that oxytocin stimulates lordosis behavior in female rats might indicate oxytocin mediates secondary action to estrogen [41]. Further mediation of the oxytocin effect by prostaglandin E2 and GnRH signaling cascade has been shown [44]. The vlVMH as well as the adjacent neuropil are extremely rich in oxytocin binding sites, in addition to the medial POA which express smaller but definite oxytocin binding [45].

### Descending POA projections

**Fig. 2:**
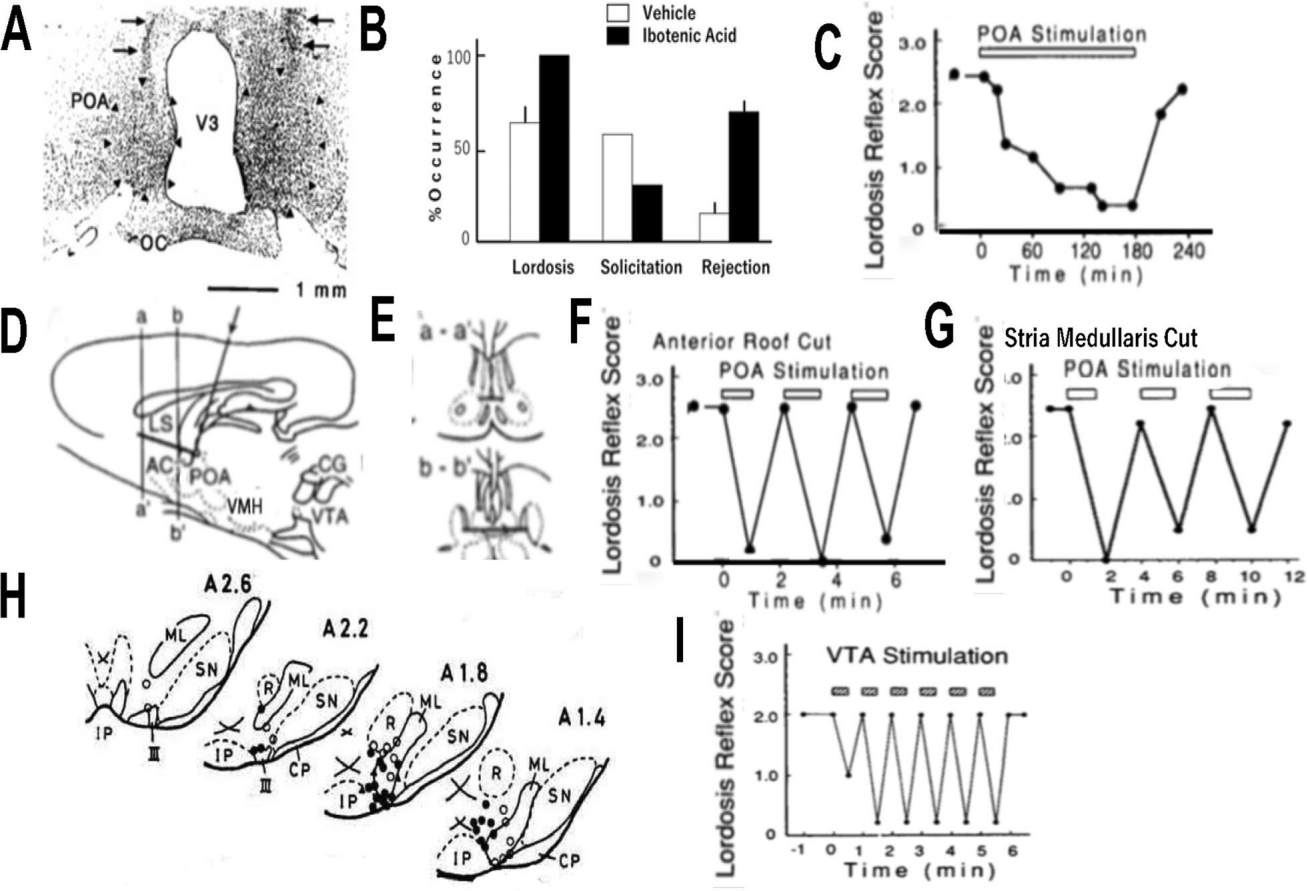
**A**. Photomicrograph of a frontal section showing ibotenic acid lesion in the medial prcoptic area (POA), immunocytochemistry against glial fibrillary acidic protein. Arrows indicate damage by the injection needles. The lesion also caused enlarged third ventricle (V3). OC, optic chiasm. **B**. Ibotenic acid lesion of the POA enhanced the lordosis quotient (percent occurrence of lordosis per 10 mounts) in ovariectomized rats given 1.5 μg estradiol benzoate. Significantly fewer animals with the lesion showed solicitatory behavior; the rejection rate (percent interruption of copulatory interactions by females) was high in animals with the lesion. Open bars, control; shaded bars, lesioned. **A** and **B**; Reprinted from [46] with permission from Elsevier. **C**. Electrical stimulation of the POA caused slow-onset, long-lasting inhibition of lordosis score (LS, an arbitrary unit with maximal dorsiflexion set at 3) in ovariectomized, estrogen-treated rats. **D**. A scheme of the anterior roof cut of the POA by rotation of an L-shaped knife. The cut disrupted dorsal input to the POA at its junction to the lateral septum (LS), dorsal to the anterior commissure (AC). **E**. The anterior roof cut of the POA in frontal sections at levels a-a' and b–b′ in D. CG, midbrain central gray; VMH, ventromedial hypotalamic nucleus; VTA, ventral tegmental area. **F**. Electrical stimulation of the POA with parameters same as in C. Note short time course in both onset and recovery of the inhibition. **G**. Bilateral disruption of the stria medularis was as effective as the frontal roof cut to cause POA-bound prompt inhibition of lordosis. **C**–**F**; Reprinted from [56] with permission from Elsevier. **H**, Stimulation sites in the VTA; filled circles, suppression of lordosis at currents below 50 μA; filled triangles, above 50 μA; open circles minor or no effect. Numbers denote distance from interaural line. *CP* cerebral peduncle, *IP* interpeduncular nucleus, *ML* medial lemniscus, *P* pons, R red nucleus, *SN* substantia nigra, *III* oculomotor nerve. VTA Stimulation caused prompt suppression of lordosis **I**. Reprinted from [63] with permission from Elsevier

A well circumscribed, homogeneous lesion of the POA produced by local infusion of ibotenic acid enhanced lordosis and precipitated reductions in the proceptivity of estrogen-treated ovariectomized female rats [46] (Fig. 2A). The dose of estrogen which induced the receptivity in the lesioned rats was much smaller than that required in the sham-operated animals (Fig. 2B). The reduced proceptivity was characterized by the loss of behavioral sensitivity to estrogen. Ibotenic acid has been described as having several practical advantages over other excitotoxins in producing homogeneous lesions over a relatively large area, leaving fibers of passage unaffected [47]. The failure of prepubertal infusion of kainic acid into the POA to alter sexual behavior in non-ovariectomized rats has been ascribed to functional compensation, because, at the time of behavioral observation several weeks after the infusion, estrous cyclicity was present in the infused animals [48]. In the male rats, ibotenic acid lesion of the POA abolishes sexual motivation [49], but promotes the lordosis [50]. The decrease in the amount of estrogen needed to induce lordosis in the lesioned animals suggests that ibotenic acid removed POA neurons that would otherwise be inhibited by estrogen. Indeed, electrical stimulation of the POA suppresses lordosis (Fig. 2C), and electrophysiological recordings have shown that estrogen mostly inhibits neuronal activity in the POA [51]. In female rats carrying Electrical stimulation of the area of the ibotenic acid lesion, which activated fibers of passage with origins in the septum or cingulate cortex [52, 53]. The female rat POA contains many neurons that are targets of estrogen [54, 55]. With the dorsal inputs to the POA removed by anterior roof cut (Fig. 2D, E) [56], electrical stimulation of the deafferented POA caused prompt interruption of lordosis (Fig. 2F) without affecting proceptive behavior. Selective disruption of the stria medularis, which carries POA inputs from the amygdala, was effective as the anterior roof cut to interrupt lordosis (Fig. 2G). The removal of POA projections to the ventral tegmental area (VTA) which continues to midbrain regions [57, 58], disinhibited the receptivity. Behavioral effects of axons of passage in the POA [59] that were spared by the excitotoxin lesion, were examined by combined ibotenic acid lesion and focal stimulation of the POA. The neurotoxic effect of ibotenic acid is expressed postsynaptically via NMDA receptor [60], that has been found in large numbers in the forebrain [61]. POA infusion of an NMDA antagonist interrupted the lordosis in fully receptive females [62]; a reduced excitatory amino acid transmission to GnRH neurons has been associated with this effect.

### The VTA

VTA stimulation in freely moving, estrogen-primed ovariectomized female rats caused a rapid and strong suppression of lordosis in response to either male mounts or manual cutaneous stimuli (Fig. 2H, I) [63]. The interruption occurred in a graded manner to increased stimulus intensity, with a low threshold. After the termination of electrical stimulation, lordosis performance returned promptly to the prestimulation level. No aversive response accompanied the blockade of lordosis. Electrical stimulation specifically blocked lordosis, without disrupting the preceptive behavior.

The POA contains a separate pool of neurons that promote proceptivity in addition to those that inhibit the receptivity. In particular, the excitability of POA neurons with axons to the VTA diminishes following estrogen in a sexually dimorphic pattern, in parallel to the capability of this steroid to induce the receptivity [16, 17].

The VTA is one of the major terminal fields of estrogen-concentrating POA neurons [64]. Electrical stimulation of axons in passage in the POA, which survived ibotenic acid lesion of the area [56] differed that the stimulation interrupted lordosis without inducing any proceptivity, whereas that of the deafferented POA [56] or the VTA [63] specifically blocked lordosis while preserving the proceptive components of female rat sexual behavior. Such dissociation of receptive and proceptive components of female sexual behavior also follows lesion or stimulation of several other brain structures [65, 66]. Among others, Whitney [67] showed that the higher lordosis quotients in female rats with POA lesion diminish if the females were allowed to evade male partner. The female could control her proximity to males. It appears that the POA contains a neural substrate for the promotion of proceptivity in addition to the suppression of lordosis. Large POA lesions or those including the rostral periventricular POA [68, 69] might also diminish lordosis through the destruction of local GnRH neurons or their axons [70]. ER positive neurons in the rostral periventricular POA [71] have been implicated in motivation of female mice sexual behavior through their innervation of GnRH neurons by kisspeptin [72].

The inhibition of proceptivity and receptivity obtained by electrical stimulation of focus of ibotenic acid lesion in the POA is due to the activation of fibers of passage which survived the excitotoxin challenge. In the lesioned animals, further transection to remove the POA afferents nullified the focal stimulation effect. The transaction was similar as the dorsal deafferentation of the POA by [73] which facilitated lordosis in non-lesioned animals (Fig. 2F, G). In the non-lesioned animals, electrical stimulation of the deafferented POA, which presumably activated local efferents, augmented the behavioral suppression through elimination of inputs via stria terminalis [56]. Thus, the POA is heterogenous not only in neuronal composition but also in axons penetrating the structure. The heterogeneity of the POA might have contributed to discrepancies among the reported POA effects on female rat sexual behavior.

### The septum

The inhibitory effect of the septum on the lordosis reflex has been recognized [73, 74]. The basic organization of midbrain projections out of the lateral septum [75] is remarkably like that of the POA, and a massive projection of the septum projects caudally to the midbrain to end in the CG, VTA, dorsal and median raphe, and laterodorsal tegmental nucleus [76, 77]. Some of these midbrain structures have been implicated in the inhibitory regulation of the lordosis reflex [57, 63, 78]. The lateral septum and the POA also resemble each other in that both contain ER-positive neurons [55]. The qualitatively similar behavioral consequences of the deletion of local efferent neurons in the POA and the removal of efferent of the septum [56] present a coherent picture of the roles of the POA and septum in the regulation of lordosis as far as they are currently understood. In conclusion, the POA contains neurons that tonically inhibit the lordosis.

### Motivational behavior in female rats

While anestrous female rats consistently avoid males, females in estrous approach and provoke males to initiate mounting. The series of affiliative or solicitatory behavior in females includes peculiar pattern of increased locomotor activity often called hopping and darting in front of the males. Increased locomotor activity in female rats in estrus embodies enhanced sexual motivation [79–81]. Estrogen action on ERα in the POA is responsible for the increased locomotion [82]. Subsets of ERα-positive POA neurons other than those involved in the inhibition of lordosis enhance locomotion. Behaviors such as hopping and darting have been used as indices of sexual motivation in females, as have other patterns of behavior such as seeking proximity to a sexually active male [66, 83, 84], the estrous female exhibits a sequence of events consisting of approach toward, orientation to, and rapid run away from the male [85]. The spinal stepping mechanism that induces locomotion is regulated by the midbrain locomotor region (MLR) in the rat [86], as originally shown in the cat [87]. In the rat, the MLR has been identified in the rostral part of the pedunculopontine and cuneiform nuclei of the midbrain [88]. Estrogen is responsible for the increased locomotion in female rats in estrus, because systemic administration of estrogen to ovariectomized rats increases both open-field [89] and wheel-running [90] activities.

The medial POA has been positively identified as a site for estrogen-induced activation of wheel running [91] while the brain site for the action of estrogen on the open-field activity [89, 92] has not been singled out. This may be due to the fact that the open-field activity is confounded by multiple factors, such as fear and emotionality. The preoptic area contributes to the rostro-caudal neural axis for the locomotor synergy [93] with its heavy projections to the MLR [88, 94]. The preoptic locomotor region [95] from which stepping can be initiated by chemical [96] or electrical [95] stimulation, is in the medial portion of the lateral POA. In contrast, the locomotor activity can be consistently reduced by cholinergic activation of the periventricular POA [97, 98]. Estrogen excites axons in the MLR with origins in the lateral POA, but inhibits those from the medial POA [99]. In the light of electrical and chemical stimulation of the POA, such diametric effects of estrogen would culminate in enhanced wheel running.

### Estrogen action to alter axonal excitability

The opposite effects of estrogen on the medial and lateral POA neurons are reminiscent of our other observations. Estrogen decreased antidromic activation thresholds in axons in the CG with origins in the VMH [100], but increased the thresholds in POA axons in the VTA [17]. The effects of estrogen on the axonal excitability are sex specific [101] and can be modified by neonatal endocrine treatments [17, 100].

Estrogen-induced changes in the axonal excitability occur in long time-courses, which implicate the involvement of receptor-mediated, genomic activation [102]. Both the medial and lateral POA contain neurons that express ER [54, 103]. Estrogen promoted the activity of Na^+^,K^+^-transporting adenosine triphosphatase in the medial basal hypothalamus while it suppressed the activity in the POA [104]. The effects were also sex specific [105]. This enzyme can reach axonal terminals by axonal flow and alter its excitability [106]. In rat uterus, estrogen alters expression of K^+^ channels [107]. Estrogen activates phosphatidyl inositol pathway which in turn can increase protein kinase C activity, Ca^2+^ mobilization, and arachidonic acid metabolism [108]. With whole-cell patch clamp study in the GT1-7 cells, we have shown that estrogen at physiological concentrations augments K(Ca) currents via ERβ, at least partly by increasing the transcription of BK channel genes, thereby modifying the cellular excitability. A large part of the K(Ca) currents is likely to comprise BK currents [109].

## Conclusion

Sexual behavior is a multitiered set of components which occur sequentially under the influence of sex hormones, particularly estrogen in female rodents. This characteristic has been thoroughly utilized in studies to identify and analyze neural mechanism for the behavior. Thus, separate neuronal circuitries, inter alia, those that promote or suppress each of proceptive and receptive component of sexual behavior have been shown. This multiplicity contributed to confusions in the interpretation of the results of studies which employed focal brain stimulation or lesion. For example, systemic administration of subthreshold dose of estrogen was needed to obtain enhanced lordosis from local implants of estrogen crystals in the VMH [8] or electrical stimulation of the VMH [9]; large amount of estrogen reinstituted lordosis in female rats with VMH lesions [18]. The latter must be due to estrogen-induced removal of inhibitory POA effects. The failure to remove estrogen-dependent POA inhibition might have caused suppressed sexual receptivity in ovariectomized rats with estrogen implants in the VMH [110]. The series of research results presented here indicate that the study of sex differences and sexual differentiation of the brain, which has been focused on morphology, has reached a stage where physiological and molecular biological approaches, such as neuronal channel expression and neurotransmitter dynamics, are required. Further developments in animal study in the field may help enhance understanding of human sexuality.

## Data Availability

Extended list of the author’s original publications can be found on online at http://sakuma-physiol.jp/cv06.pdf.
